# Elevated Procalcitonin Levels in a Patient With Diabetic Ketoacidosis in the Absence of Infection

**DOI:** 10.7759/cureus.24154

**Published:** 2022-04-15

**Authors:** Basheer Mohammed, Anass Dweik, Ola Al-Jobory, Kelly Mcmaster

**Affiliations:** 1 Internal Medicine, Texas Tech University Health Sciences Center, Amarillo, USA

**Keywords:** clinical evaluation of diabetic ketoacidosis, sepsis, laboratory markers, procalcitonin, diabetic ketoacidosis

## Abstract

Bacterial infections are one of the major precipitating factors in diabetic ketoacidosis (DKA). Procalcitonin (PCT) is highly specific in identifying bacteria sepsis, but PCT may be elevated in patients who do not have sepsis. Here, we report a 25-year-old patient admitted to the ICU for DKA. Initial laboratory investigation showed elevated PCT of 0.87 ng/ml and reached a concentration of 15.88 ng/ml on the second day of admission. PCT levels trended down to 4.7 ng/ml by the third day of admission. This case report shows PCT levels can be increased in patients with DKA even in the absence of infection and PCT levels decrease with clinical improvement of DKA without administration of antibiotics.

## Introduction

Diabetic ketoacidosis (DKA) is one of the most common, acute, and major life-threatening complications of diabetes mellitus. The most common causes of DKA are underlying or concurrent infection, missed or interrupted insulin treatment, newly diagnosed, previously unknown diabetes, and a high level of medical, surgical, and emotional stress [1].

Infection is the most frequent trigger [2]. It is known that DKA is associated with leukocytosis and an increase in acute-phase proteins, such as C-reactive protein, tumor necrosis factor-alpha, interleukin-1 beta, and interleukin-6, all of which are usually increased in bacterial infections as well [3]. Procalcitonin (PCT) levels increase in bacterial infection/sepsis, and they are useful markers of bacterial infections [4].

It has been reported that PCT levels can be dramatically elevated without any occult focus of infection in patients with DKA and PCT levels decrease in concentrations with the improvement of DKA even without antibiotic therapy [5,6]. Thus, one cannot simply infer infection as the trigger; this reality combined with the need for antibiotic stewardship mandates that empiric antibiotic therapy not be an automated or knee-jerk part of DKA management.

## Case presentation

A 25-year-old Caucasian male presented to the hospital with a reduced level of consciousness and confusion. Symptoms developed a few hours before admission. One year earlier, he was diagnosed with type 1 diabetes and his glycemic regimen consisted of neutral protamine Hagedorn (NPH) 20 IU in the morning and 10 IU at night, and regular insulin before meals. The patient was noncompliant with his medications, diet, and glycemic monitoring. He also had a history of methamphetamine use, although he denied any recent use. Initial physical examination showed a temperature of 98°F, a heart rate of 106 beats per minute, blood pressure of 150/80 mmHg, and respiratory rate of 34 breaths per minute. The patient was disoriented and confused, with dry mucous membranes. Bilateral non-pitting pedal edema was noted up to the distal 1/3rd of the leg. The remainder of the exam was benign/nonsignificant. Initial laboratory tests showed hyperglycemia with a blood glucose level of 987 mg/dL (normal: 74-106 mg/dL).

Full laboratory workup (Table 1) was significant for severe anion gap metabolic acidosis, hyperkalemia, hypochloremia, hyponatremia, lactic acidosis, ketosis with elevated acetone levels, leukocytosis, and prerenal acute kidney injury. Urine toxicology was positive for amphetamines. Urinalysis was negative for pyuria, leukocyte esterase, and nitrite. Chest radiograph showed no focal infiltrate or consolidation. EKG showed tall, tented T waves in almost all leads consistent with hyperkalemia. Blood cultures were drawn. The initial PCT level was 0.87 ng/ml (normal < 0.075 ng/mL). The patient was diagnosed with DKA and resuscitated with normal saline, electrolyte replacement, and continuous insulin infusion with close glycemic monitoring.

**Table 1:** Laboratory results

	Reference range	Day 1	Day 2	Day 3
Procalcitonin	<0.075 ng/ml	0.87	15.88	4.77
White blood cell count	4-10.6 x 10e3/mcL	17.6	10.2	6.0
Neutrophils	40-76%	82.6	77.2	65.2
Hemoglobin	14.5-17.7 gm/dL	11.8	11.2	11.3
Platelet count	150-400 x 10e3/mcL	333	109	226
International normalized ratio	0.89-1.07	1.18		
Sodium	136-145 mmol/L	126	137	141
Potassium	3.5-5.1 mmol/L	6.9	4.5	3.8
Chloride	98-107 mmol/L	92	104	114
Bicarbonate	21-32 mmol/L	3	23	26
Anion gap	9-18 mmol/L	31	17	7
Lactic acid	0.4-2 mmol/L	5.2	0.7	
Creatinine	0.6-1.3 mmol/L	1.6	0.9	0.6
Lipase	73-393 units/L	40		
Acetone		1:32		
Arterial PH	7.35-7.45	6.89		
Arterial partial pressure of carbon dioxide (PC02)	35-45 mmHg	11.7		
Arterial partial pressure of oxygen (PO2)	80-105 mmHg	125		

Repeat PCT on day two was elevated at 15.880 ng/mL (normal < 0.075 ng/mL). The patient did not have any nausea, vomiting, abdominal pain, confusion, fever, or chills. Vital signs were normal, and no clinical evidence of infection was noted. C-reactive protein was 8.22 mg/L (normal < 3 mg/L). Blood cultures were negative, and an echocardiogram showed normal ejection fraction and normal valves with no vegetations. Lower extremity duplex ultrasound was negative for deep venous thrombosis. Due to antibiotic stewardship considerations, and the perception that ICU surveillance can reveal all signs of acute sepsis very quickly with good treatment outcomes, the patient did not receive any antibiotics and PCT spontaneously trended down on day three to 4.7 ng/mL (normal < 0.075 ng/mL). He was discharged home on day three after blood glucose stabilized on 70/30 insulin (this regimen was chosen due to its low cost, as the patient was uninsured).

## Discussion

PCT is a serum biomarker of an inflammatory response, including but not limited to systemic bacterial infection. It is produced within the C-cells of the thyroid gland as a prohormone of calcitonin, which is intracellularly cleaved by proteolytic enzymes into the active hormone. In healthy patients, PCT levels are undetectable [7]. PCT production is induced in most tissues, especially the lungs and intestine, and released into the blood when systemic inflammation is due to bacterial infections. Bacteria endotoxins [8] and cytokines including tumor necrosis factor (TNF)-alpha, interleukin-1-beta, and interleukin-6 stimulate the synthesis of PCT [9-11]. Leukocytes do not play an important role in PCT production during sepsis [12]. PCT levels can also increase in non-infectious conditions that elevate cytokine levels such as burns, trauma, surgery, and pancreatitis [13].

Anno et al. demonstrated that PCT levels are elevated in patients with type 1 diabetes mellitus in the setting of DKA, and increased PCT levels were hypothesized to constitute a biomarker of DKA without infection [14]. Although we do not know why PCT levels are elevated under DKA conditions, it has been speculated that metabolic cytokine storms and some signal transductions are associated with such elevations; however, the exact mechanism remains unknown [15]. Ivaska et al. reported that PCT may show a considerable elevation in children with DKA without an invasive bacterial infection. PCT levels correlated with the severity of hyperglycemia and acidosis. It was theorized that this was related to the hyperlactatemia associated with DKA and/or elevated levels of tumor necrosis factor and other cytokines [16]; the relevance of these observations and syntheses to our adult patient is uncertain.

PCT can be increased in patients with DKA, as noted in our patient, and care needs to be taken in the interpretation of this result to prevent unnecessary investigations and unwarranted antibiotic administration if no signs of infection are present, provided one is sufficiently confident of close monitoring and adequate support. This decision is not easy and requires substantial confidence in one’s diagnostic skill, but the avoidance of antibiotics when not needed means not only reduced expense but also less selection of resistant strains, less alteration of native flora, and reduced risk of antibiotic-related complications such as *Clostridium difficile* infections.

## Conclusions

PCT levels can be increased in patients with DKA. The role of PCT within the context of DKA warrants further investigation because it may not always indicate bacterial infection. Being cognizant of this can help promote antibiotic stewardship, reduce antibiotic exposure, and development of resistance. Several studies have reported that PCT can be increased in DKA even in the absence of bacterial infection. However, the mechanism by which DKA leads to elevated PCT levels remains unknown.

## References

[1] (2022). Diabetic ketoacidosis (DKA). https://emedicine.medscape.com/article/118361-overview#a5.

[2] Ahmed A, Rahim M, Rahman M, Nazim RF, Uddin KN (2014). Diabetic ketoacidosis: pattern of precipitating causes. J Enam Med Coll.

[3] Karavanaki K, Karanika E, Georga S (2011). Cytokine response to diabetic ketoacidosis (DKA) in children with type 1 diabetes (T1DM). Endocr J.

[4] Simon L, Gauvin F, Amre DK, Saint-Louis P, Lacroix J (2004). Serum procalcitonin and C-reactive protein levels as markers of bacterial infection: a systematic review and meta-analysis. Clin Infect Dis.

[5] Cipriano A, Rebelos E, Park N, Santini M (2018). Moderate increase of serum levels of procalcitonin in diabetic ketoacidosis. Neth J Med.

[6] Aksu NM, Aksoy DY, Akkaş M (2013). 25-OH-vitamin D and procalcitonin levels after correction of acute hyperglycemia. Med Sci Monit.

[7] Maruna P, Nedelníková K, Gürlich R (2000). Physiology and genetics of procalcitonin. Physiol Res.

[8] Dandona P, Nix D, Wilson MF, Aljada A, Love J, Assicot M, Bohuon C (1994). Procalcitonin increase after endotoxin injection in normal subjects. J Clin Endocrinol Metab.

[9] Becker KL, Nylén ES, White JC, Müller B, Snider RH Jr (2004). Clinical review 167: procalcitonin and the calcitonin gene family of peptides in inflammation, infection, and sepsis: a journey from calcitonin back to its precursors. J Clin Endocrinol Metab.

[10] Assicot M, Gendrel D, Carsin H, Raymond J, Guilbaud J, Bohuon C (1993). High serum procalcitonin concentrations in patients with sepsis and infection. Lancet.

[11] Harbarth S, Holeckova K, Froidevaux C (2001). Diagnostic value of procalcitonin, interleukin-6, and interleukin-8 in critically ill patients admitted with suspected sepsis. Am J Respir Crit Care Med.

[12] Monneret G, Laroche B, Bienvenu J (1999). Procalcitonin is not produced by circulating blood cells. Infection.

[13] Christ-Crain M, Müller B (2005). Procalcitonin in bacterial infections--hype, hope, more or less?. Swiss Med Wkly.

[14] Anno T, Shigemoto R, Kawasaki F, Irie S, Miyashita N, Kaku K, Kaneto H (2020). Marked elevation of plasma procalcitonin levels in patients with diabetic ketoacidosis: a possible useful diagnostic biomarker. Diabetes Metab.

[15] Linscheid P, Seboek D, Nylen ES (2003). In vitro and in vivo calcitonin I gene expression in parenchymal cells: a novel product of human adipose tissue. Endocrinology.

[16] Ivaska L, Elenius V, Mononen I, Ruuskanen O, Peltola V (2016). Discrepancies between plasma procalcitonin and C-reactive protein levels are common in acute illness. Acta Paediatr.

